# Membrane-type 1 matrix metalloproteinase-mediated progelatinase A activation in non-tumorigenic and tumorigenic humaneratinocytes

**DOI:** 10.1054/bjoc.2000.1454

**Published:** 2000-10-26

**Authors:** P Baumann, P Zigrino, C Mauch, D Breitkreutz, R Nischt

**Affiliations:** Department of Dermatology, University of Cologne, Köln, 50924; Division of Carcinogenesis and Differentiation, German Cancer Research Center, Heidelberg, 69120, Germany

**Keywords:** type IV collagenases, activation, keratinocytes, HaCaT-*ras*, cell–matrix interactions

## Abstract

Elevated expression of type IV collagenases (MMP-2 and MMP-9) has been strongly correlated with tumour progression and metastasis in various tumours. Here, we analysed expression and activation of these MMPs in non-tumourigenic HaCaT cells and the malignant HaCaT variant II-4 _rt_. In monolayer cultures, both cell types secreted latent MMP-2 (proMMP-2) in comparable amounts, while MMP-9 production was clearly higher in II-4 _rt_ cells. Upon contact with fibrillar collagen type I the malignant II-4 _rt_ cells, but not the HaCaT cells, gained the capability to activate proMMP-2. This process is shown to be membrane-associated and mediated by MT1-MMP. Surprisingly, all membrane preparations from either HaCaT cells or II-4 _rt_ cells grown as monolayers, as well as within collagen gels, contained considerable amounts of active MT1-MMP. However, within collagen gels HaCaT cells showed significantly higher TIMP-2 levels compared to II-4 _rt_ cells. This indicates that TIMP-2 might play a central role for MT1-MMP-mediated gelatinolytic activity. Indeed, collagen type I-induced MT1-MMP-mediated proMMP-2 activation by II-4 _rt_ membranes could be completely abolished by an excess of TIMP-2. In conclusion, our data suggest that MT1-MMP-mediated proMMP-2 activation might be associated with malignant progression of epidermal tumour cells. © 2000 Cancer Research Campaign


*British Journal of Cancer* (2000) **83**(10), 1387–1393
© 2000 Cancer Research Campaign
doi: 10.1054/ bjoc.2000.1454, available online at http://www.idealibrary.com on IDEAL®

### Membrane-type 1 matrix metalloproteinase-mediated progelatinase A activation in non-tumorigenic and tumorigenic human keratinocytes

**P Baumann[1], P Zigrino[1], C Mauch[1], D Breitkreutz[2] and R Nischt[1]**

[1]Department of Dermatology, University of Cologne, 50924 Köln; [2]German Cancer Research Center, Division of Carcinogenesis and Differentiation, 69120 Heidelberg, Germany

**Summary** Elevated expression of type IV collagenases (MMP-2 and MMP-9) has been strongly correlated with tumour progression and metastasis in various tumours. Here, we analysed expression and activation of these MMPs in non-tumourigenic HaCaT cells and the malignant HaCaT variant II-$4_{rt}$. In monolayer cultures, both cell types secreted latent MMP-2 (proMMP-2) in comparable amounts, while MMP-9 production was clearly higher in II-$4_{rt}$ cells. Upon contact with fibrillar collagen type I the malignant II-$4_{rt}$ cells, but not the HaCaT cells, gained the capability to activate proMMP-2. This process is shown to be membrane-associated and mediated by MT1-MMP. Surprisingly, all membrane preparations from either HaCaT cells or II-$4_{rt}$ cells grown as monolayers, as well as within collagen gels, contained considerable amounts of active MT1-MMP. However, within collagen gels HaCaT cells showed significantly higher TIMP-2 levels compared to II-$4_{rt}$ cells. This indicates that TIMP-2 might play a central role for MT1-MMP-mediated gelatinolytic activity. Indeed, collagen type I-induced MT1-MMP-mediated proMMP-2 activation by II-$4_{rt}$ membranes could be completely abolished by an excess of TIMP-2. In conclusion, our data suggest that MT1-MMP-mediated proMMP-2 activation might be associated with malignant progression of epidermal tumour cells. © 2000 Cancer Research Campaign

**Keywords**: type IV collagenases; activation; keratinocytes; HaCaT-*ras*; cell–matrix interactions

Local degradation of connective tissue in the vicinity of the cell surface is thought to play an essential role for tumour cell invasion and metastasis. Degradation of extracellular matrix components is accomplished through an array of proteolytic enzymes including members of both the matrix metalloproteinase (MMP) and serine protease families (Birkedal-Hansen et al, 1993; Stetler-Stevenson et al, 1993; Yu et al, 1997).

Among MMPs, the type IV collagenases MMP-2 and MMP-9 are considered to play a critical role in tumour progression. Elevated expression and activation of MMP-2 and MMP-9 have been strongly correlated with the invasive phenotype of tumours like squamous cell carcinomas (Pyke et al, 1992; Kusukawa et al, 1993), gastric (Brown et al, 1990), and breast (Okada et al, 1995) carcinomas.

Like most MMPs, MMP-2 (gelatinase A, 72-kDa type IV collagenase) is secreted as an inactive zymogen that has to be activated by removal of the N-terminal propeptide. In vitro, serine proteases have been shown to activate most MMPs (Birkedal-Hansen et al, 1993). However, MMP-2 is unique among the MMPs in that its activation is achieved in a membrane-associated manner by membrane-type matrix metalloproteinases (MT-MMPs), such as MT1-MMP (Sato et al, 1994). MT1-MMP (MMP-14) is also synthesized as a proform which can be activated both intracellularly by the serine protease furin (Pei and Weiss, 1996), and extracellularly by plasmin (Okumura et al, 1997) and urokinase-type plasminogen activator (Kazes et al, 1998). Although five different membrane-bound MMPs have been described thus far (Sato et al, 1994; Takino et al, 1995; Will and Hinzmann, 1995; Puente et al, 1996; Butler et al, 1997; Pei, 1999), among them MT2-, MT3-, and MT5-MMP have been reported to activate proMMP-2, MT1-MMP is the one with the best documented correlation to the invasive phenotype of different types of cancer (Okada et al, 1995; Tsunezuka et al, 1996; Gilles et al, 1997; Nakamura et al, 1999). MT1-MMP production or activation has been shown in vitro to be up-regulated by fibrillar type I collagen (Azzam and Thompson, 1992; Seltzer et al, 1994; Haas et al, 1998; Kurschat et al, 1999), an interaction which is likely to occur in vivo when tumour cells invade the surrounding stroma. The activation of proMMP-2 by MT1-MMP has been reported to be dependent on the presence of low amounts of the tissue inhibitor of matrix metalloproteinases (TIMP)-2, which is required as a bridging molecule for the formation of a membrane-bound activation complex composed of MT1-MMP, TIMP-2 and latent MMP-2 (Strongin et al, 1995; Butler et al, 1998). At high concentrations, however, TIMP-2 has been shown to inhibit proMMP-2 activation, presumably by blocking the activity of MT1-MMP (Will et al, 1996; Itoh et al, 1998; Zucker et al, 1998).

The spontaneously immortalized non-tumorigenic human keratinocyte cell line HaCaT (Boukamp et al, 1988) and the *c-ras*$^{Ha}$ transfected HaCaT clones (Boukamp et al, 1990) provide an excellent model to study cellular changes associated with malignant progression of epithelial cells. While HaCaT cells reveal a nearly normal epidermal phenotype upon transplantation onto athymic nude mice (Breitkreutz et al, 1998), the malignant variants of the *c-ras* clones grow invasively and form squamous

*Received 11 May 2000*
*Revised 12 July 2000*
*Accepted 18 July 2000*

*Correspondence to:* R Nischt cell carcinoma-like tumours showing severe alterations at the cell-matrix interface (Tomakidi et al, 1999). In the present study, we analysed MMP-2 and MMP-9 expression in HaCaT cells and the *c-ras*$^{Ha}$ transfected HaCaT clone II-4$_{rt}$ to determine whether alterations in the production of these MMPs are associated with acquisition of the malignant phenotype.

### MATERIALS AND METHODS

### Cells and and culture conditions

HaCaT cells, a non-tumorigenic keratinocyte cell line and the *c-ras*$^{Ha}$ transfected tumourigenic HaCaT cell clone II-4$_{rt}$ were kindly provided by Dr N Fusenig (German Cancer Research Center, Heidelberg, Germany). Both cell lines were routinely cultured in Dulbecco's modified Eagle medium (DMEM) supplemented with 10% fetal calf serum (FCS), 2 mM glutamine and 100 U ml$^{-1}$ each of penicillin and streptomycin. Three-dimensional collagen type I lattices were prepared as described previously (Mauch et al, 1988). Briefly, $4 \times 10^5$ cells ml$^{-1}$ were seeded into collagen gels containing 2.4 mg ml$^{-1}$ bovine collagen type I (Cellon, Cell Systems, Germany).

### Preparation of conditioned media

Cells were cultured as monolayers on collagen type I-coated dishes (10 µg cm$^{-2}$) or within collagen gels with daily medium changes. At indicated time-points the cultures were washed with phosphate buffered saline (PBS) and the medium replaced by serum-free DMEM. 24 h later the media were collected, the cells trypsinized, or released from the collagen gels by treatment with bacterial collagenase D (1 mg ml$^{-1}$) and counted. Alternatively, conditioned media and cells grown as monolayers or in collagen gels were combined and homogenized by sonication. For inhibition experiments the cell suspensions were preincubated for 30 min with the synthetic furin inhibitor Decanoyl-Arg-Val-Lys-Arg-chloromethylketone (CMK; Bachem Biochemicals, Germany), with the CMK solvent methanol, with aprotinin (ROTH, Germany), or with 1,10 phenanthroline (Sigma, Germany) and then seeded into collagen gels. The inhibitors were added at the following concentrations: CMK = 25 and 50 µM; aprotinin = 10 µM; 1,10 phenanthroline = 10 and 20 µM. The peptides UKI and ni68 (20 µg ml$^{-1}$) inhibiting uPA and interaction of uPA with its receptor, respectively, were also included. Both inhibitors were kindly provided by Dr V Magdolen (Department of Gynaecology, Technical University, Klinikum rechts der Isar, Munich, Germany). After 24 h the cultures were washed with PBS and further cultivated for 24 h in serum-free DMEM containing the inhibitor. Then the culture samples were processed as described before.

### Gelatin zymography

For gelatin zymography supernatants corresponding to $1.5 \times 10^4$ cells or 10 µl homogenates of combined media and cells were separated on non-reducing 10% SDS polyacrylamide gels containing 1 mg ml$^{-1}$ bovine gelatin (Sigma, Germany) (Herron et al, 1986). After electrophoresis the gels were washed in 2.5% Triton X-100 for 30 min and then incubated in enzyme substrate buffer (50 mM Tris-HCl, pH 8.5, 5 mM $CaCl_2$) overnight at 37°C. Gels were stained with Coomassie Blue R250 and destained in water.

### Preparation of crude membranes

For preparation of crude plasma membranes the collagen gels were disrupted by mechanical shearing through a 10 ml syringe and subsequent incubation with bacterial collagenase D (1 mg ml$^{-1}$) for approximately 10 min until no visible collagen fibres were left. After addition of half a volume of FCS the cells were pelleted (2000 ***g*** for 5 min at 4°C) and resuspended in PBS containing 1 mM Pefabloc (Boehringer Mannheim, Germany), 10 µg ml$^{-1}$ aprotinin and 1 µg ml$^{-1}$ leupeptin (Sigma, Germany). After cell lysis by three cycles of freezing in liquid nitrogen and thawing, the lysate was homogenized by sonification. The plasma membranes were pelleted by centrifugation (150 000 ***g*** for 30 min at 4°C) and resuspended in PBS. The protein concentration was determined using a commercial assay (Bio-Rad, Germany). For activity assays 10 µg of the membrane preparations were incubated overnight at 37°C with 20 µl fibroblast-conditioned medium containing proMMP-2. Incubations were performed in the presence of 1, 5 or 10 µg of anti-MT1-MMP peptide antibodies blocking specifically MT1-MMP-mediated proMMP-2 activation (raised against a peptide corresponding to the residues 160–173 of human MT1-MMP, clone 114–1F2, Fuji Chemicals, Japan) or in the presence of 3.8 or 7.6 nM recombinant TIMP-2 (Calbiochem-Novabiochem, Germany). 5 µl of the samples were analysed by gelatin zymography.

### Northern blot analysis

Total RNA was isolated from cells grown as monolayers or within collagen gels using RNAzol™B according to the manufacturer's instruction (WAK-Chemie, Germany). 10 µg of total RNA were separated in 1% formaldehyde/agarose gels and blotted onto nylon membranes (Amersham-Pharmacia-Biotech, Germany). Filters were hybridized with $^{32}$P-labelled cDNA probes for MT1-MMP (Sato et al, 1994), MMP-2 (Collier et al, 1988), and TIMP-2 (Stetler-Stevenson et al, 1990). As a control for equal loading and the integrity of the RNA the filters were rehybridized with a $^{32}$P-labelled 18S rRNA oligonucleotide (Carlson et al, 1993).

### Immunochemical analysis

For immunoblots crude plasma membrane preparations (20 µg) were separated on 10% SDS polyacrylamide gels under reducing conditions and transferred onto nitrocellulose membranes (Amersham-Pharmacia-Biotech). After blockage with 5% non-fat milk powder in PBS/0.5% (v/v) Tween the membranes were incubated overnight with 1 µg ml$^{-1}$ of the anti-MT1-MMP peptide antibody 114–1F2 at 4°C, followed by an incubation with a horseradish peroxidase conjugated anti-mouse IgG antibody (Dako, Germany) for 1 h. Bound antibodies were detected with ECL according to the supplier's protocol (Amersham-Pharmacia-Biotech). For quantification of TIMP-2 protein levels conditioned media combined with sonicated cells or collagen gels (100 µl) were subjected to ELISA multiwell plates, precoated with anti-TIMP-2 antibodies, and measured following the supplier's instructions (Amersham-Pharmacia-Biotech). Serial dilutions of human recombinant TIMP-2 were used as internal standards.

 *© 2000 Cancer Research Campaign*

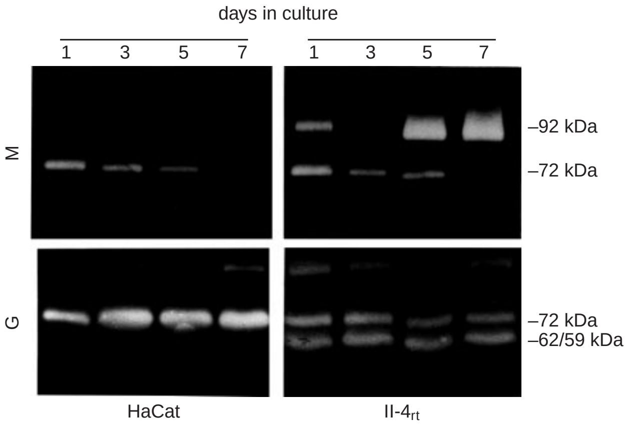

**Figure 1** Expression of type IV collagenases in non-tumorigenic HaCaT cells and the c-ras$^{Ha}$-transfected HaCaT clone II-4$_{rt}$. The two cell types were cultivated either on collagen type I coated dishes (M) or within collagen lattices (G). At days 1, 3, 5 and 7 the monolayer and collagen cultures were washed with PBS and cultures continued for 24 h in serum-free medium. Conditioned media corresponding to $1.5 \times 10^4$ cells were analysed by gelatin zymography. Positions of proMMP-9, proMMP-2 and MMP-2 are indicated by their molecular weights. The data presented here are representative of three different experiments

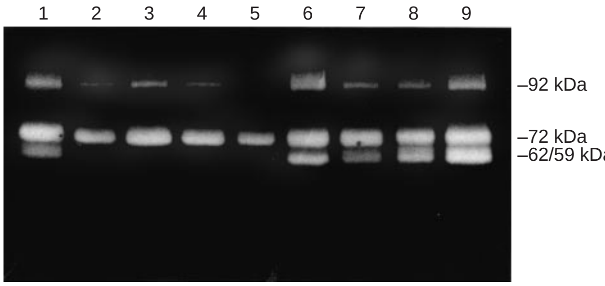

**Figure 2** Inhibition of proMMP-2 activation by II-4$_{rt}$ cells grown in collagen gels. Before seeding in collagen lattices, II-4$_{rt}$ cells were preincubated for 30 min at 37°C as follows: no inhibitor (lane 1), 1,10 phenanthroline (lane 2 = 10 μM, lane 3 = 20 μM), the furin inhibitor CMK (lane 4 = 25 μM, lane 5 = 50 μM), the CMK solvent methanol (lane 6), aprotinin (lane 7 = 20 μM) and the uPA inhibitors UK1 (lane 8 = 20 μg ml$^{-1}$) and ni68 (lane 9 = 20 μg ml$^{-1}$). After 24 h cultivation the collagen gels were washed with PBS and cultured in serum-free medium containing fresh inhibitors. After 24 h the conditioned media were combined with the collagen gels, homogenized and 10 μl of the homogenates analysed by gelatin zymography

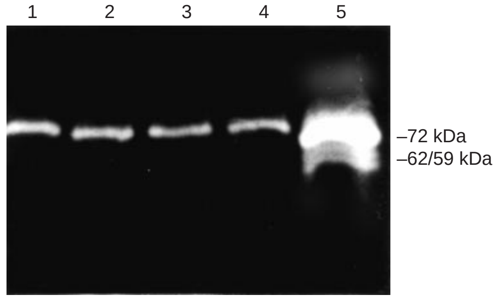

**Figure 3** Activation of exogenous proMMP-2 by crude membrane preparations from HaCaT and II-4$_{rt}$ cells. Both cell types were grown as monolayers or within collagen gels for 24 h. Then the cells were collected and crude membranes prepared as described in Materials and Methods. 10 μg of the membrane preparations were incubated (24 h at 37°C) with 20 μl fibroblast conditioned medium, containing proMMP-2. Activation of proMMP-2 was assayed by gelatin zymography. As a control fibroblast conditioned medium was applied in lane 1. The crude membrane preparations from HaCaT (lane 2) and II-4$_{rt}$ (lane 4) monolayer cultures and from HaCaT (lane 3) and II-4$_{rt}$ (lane 5) collagen gel cultures are shown. The molecular sizes (kDa) of the latent and active MMP-2 protein forms are marked to the right

### RESULTS

### Effect of cell–matrix interactions on proMMP-2 activation in non-tumorigenic HaCaT cells and in the tumorigenic HaCaT clone II-4$_{rt}$

HaCaT cells and the tumorigenic HaCaT cell clone II-4$_{rt}$ were grown as monolayers on collagen type I coated dishes or within three-dimensional fibrillar collagen lattices. On days 1, 3, 5 and 7 conditioned media were prepared and analysed by gelatin zymography (Figure 1). In monolayer cultures both cells types produced comparable amounts of MMP-2, but only in its latent form, while MMP-9 production did clearly differ, being higher in II-4$_{rt}$ cells. When cultured within a three-dimensional matrix composed of native type I collagen fibrils, HaCaT cells continued to produce the latent form of MMP-2, whereas the tumorigenic II-4$_{rt}$ cells gained the capability to convert proMMP-2 into its 62/59 kDa active forms. Surprisingly, the strong induction of proMMP-9 observed in II-4$_{rt}$ monolayer cultures was greatly reduced by growing these cells in contact with fibrillar collagen. Preincubation of II-4$_{rt}$ cells with the MMP-inhibitor 1,10 phenanthroline and the synthetic furin inhibitor CMK (Stieneke-Grober et al, 1992) resulted in complete suppression of collagen type I-induced activation of proMMP-2. The serine protease inhibitor aprotinin and the specific urokinase inhibitors did not affect this activation process (Figure 2). These observations indicate that MT-MMPs which have been shown to be activated intracellularly by furin-like protease are likely to be involved in proMMP-2 activation.

To test this, we prepared crude membrane fractions from HaCaT and II-4$_{rt}$ cells cultivated as monolayers or within collagen gels and incubated them with fibroblast conditioned medium containing proMMP-2. Only the membrane fraction isolated from II-4$_{rt}$ cells grown within the collagenous matrix showed activation of fibroblast derived proMMP-2 (Figure 3).

### MT1-MMP is constitutively expressed in HaCaT and II-4$_{rt}$ cells under both culture conditions

In order to elucidate the mechanism underlying proMMP-2 activation in II-4$_{rt}$ cells, we analysed MMP-2 and MT1-MMP expression on both transcript and protein levels. RNA analysis (Figure 4) revealed low MMP-2 mRNA levels in HaCaT and II-4$_{rt}$ cells under monolayer conditions. After contact with fibrillar collagen the transcript levels increased significantly in both cell types. Surprisingly, HaCaT cells as well as II-4$_{rt}$ cells displayed constitutive expression of MT1-MMP mRNA in monolayer cultures with higher levels found in II-4$_{rt}$ cells. In collagen gels both cell types showed an induction of MT1-MMP mRNA to comparable levels.

As shown in Figure 5, membranes purified from HaCaT cells grown as monolayers or within collagen gels for 24 h displayed two immunoreactive bands of equal intensity, with the 63 kDa band corresponding to proMT1-MMP and the 60 kDa band to the active processed form of MT1-MMP (Lehti et al, 1998; Maquoi et al, 1998). In contrast, membranes from II-4$_{rt}$ cells grown as monolayers and within the collagenous matrix contained mainly

*© 2000 Cancer Research Campaign*

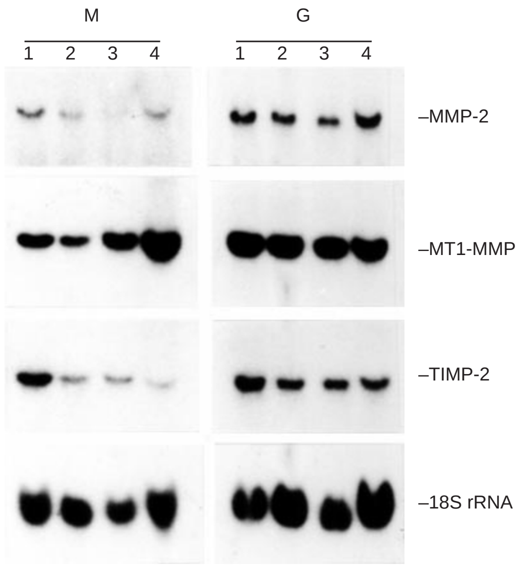

**Figure 4** Detection of MMP-2, MT1-MMP and TIMP-2 mRNA in the HaCaT clones grown as monolayers (M) or within collagen gels (G). For RNA extraction HaCaT cells (lanes 1 and 2) and II-$4_{rt}$ cells (lanes 3 and 4) were cultivated for 24 h (lanes 1 and 3) or 72 h (lanes 2 and 4). 10 µg of the RNA samples were separated in a 1% formaldhyde/agarose gel, blotted onto a nylon membrane and hybridized successively with $^{32}$P-labelled cDNA probes for MT1-MMP, TIMP-2 and MMP-2. RNA loading was assessed by hybridization with an 18S rRNA oligonucleotide. Transcript lengths are 4.5 kb for MT1-MMP, 3.0 kb for TIMP-2 and 3.1 kb for MMP-2

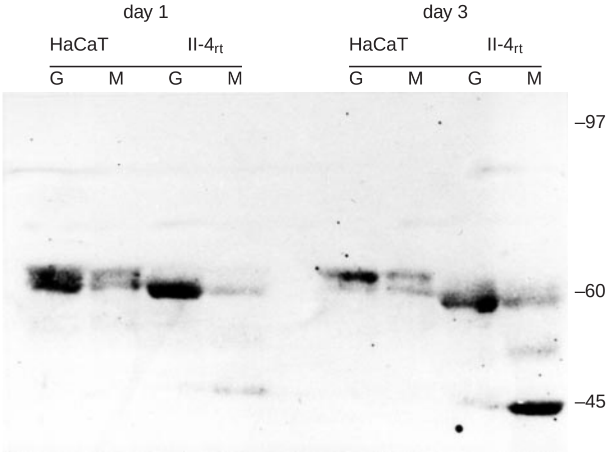

**Figure 5** Detection of latent and active MT1-MMP in crude membrane preparations isolated from HaCaT and II-$4_{rt}$ cells. Membrane fractions (20 µg) were separated on a 10% SDS/polyacrylamide gel under reducing conditions. After transfer of the proteins onto a nitrocellulose membrane MT1-MMP was detected with the monospecific peptide antibody 114-1F2 (1 µg ml$^{-1}$) followed by incubation with a horseradish peroxidase conjugated anti-mouse IgG antibody (1:2000). Bound antibodies were detected with the ECL system. Membranes were isolated from HaCaT and II-$4_{rt}$ cells cultivated for 1 or 3 days either as monolayers (M) or in collagen lattices (G). Molecular weight standards (kDa) are indicated to the left

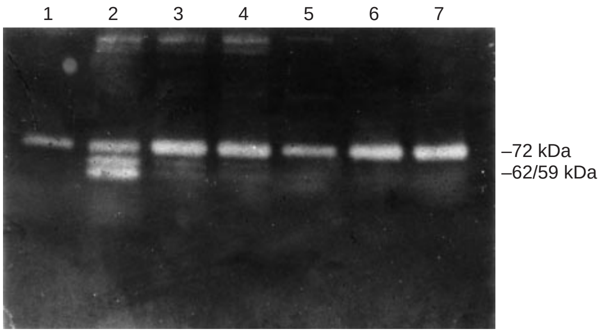

**Figure 6** Inhibition of membrane-mediated proMMP-2 activation by MT1-MMP specific antibodies and by addition of recombinant TIMP-2. 10 µg of crude membrane preparations from II-$4_{rt}$ cells grown in collagen gels for 24 h, were incubated with fibroblast conditioned medium (20 µl) containing latent MMP-2 (lane 1). The incubations were performed either in the absence (lane 2), or in the presence of 1 µg (lane 3), 5 µg (lane 4), and 10 µg (lane 5) of MT1-MMP-specific antibodies, or in the presence of 5 ng (lane 6) and 10 ng (lane 7) recombinant TIMP-2, respectively. After incubation proMMP-2 activation was assayed by gelatin zymography

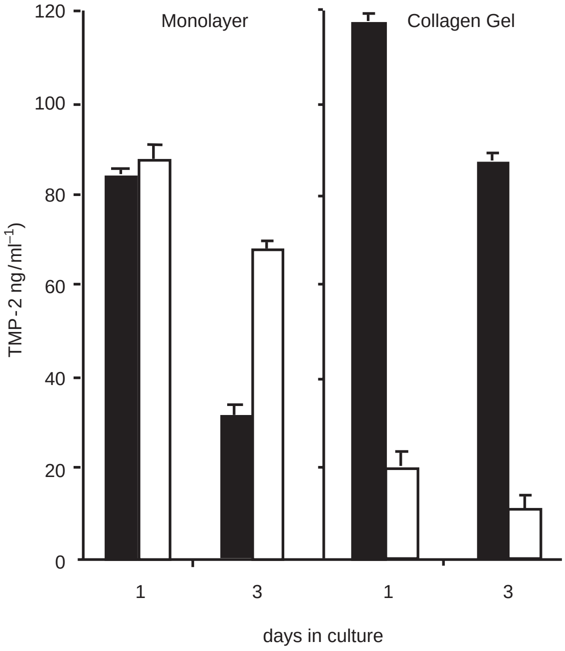

**Figure 7** TIMP-2 production by HaCaT cells and II-$4_{rt}$ cells cultivated as monolayers or within collagen gels. HaCaT (black bars) and II-$4_{rt}$ (white bars) cells were grown as monolayers and in collagen gels for 24 and 72 h. At these time-points the cultures were washed with PBS and continued in serum-free medium for a further 24 h. Then the cells/collagen gels were combined with their conditioned media and homogenized. TIMP-2 levels were determined using an ELISA as described in Materials and Methods. Each bar represents the mean ± SEM of two independent experiments performed in duplicate ($P < 0.05$)

active MT1-MMP, while proMT1-MMP was barely detectable at both time-points. Upon contact with fibrillar collagen, both cell types showed an increase of the active form. Under this condition the differences in the ratio of latent/active MT1-MMP in HaCaT and II-$4_{rt}$ cells became even more obvious. The appearance of the lower molecular weight protein band of about 45 kDa detected in some membrane fractions is likely to be caused by further processing of MT1-MMP, as described by Lehti et al (1998).

### MT1-MMP and TIMP-2 play substantial roles for proMMP-2 activation induced by fibrillar collagen in II-$4_{rt}$ cells

To test whether MT1-MMP is involved in proMMP-2 activation by II-$4_{rt}$ cells, membranes were isolated from II-$4_{rt}$ cells grown in collagen gels for 24 h and incubated overnight with fibroblast

 *© 2000 Cancer Research Campaign*

conditioned medium containing proMMP-2. Analysis by gelatin zymography revealed activation of fibroblast derived proMMP-2 to the 62/59 kDa active forms (Figure 6, lane 2, see also Figure 3). However, when the membranes were preincubated with increasing amounts of MT1-MMP-specific antibodies, binding to the catalytic center of MT1-MMP, proMMP-2 activation was completely abolished in a concentration-dependent manner (Figure 6, lanes 3–5) indicating that MT1-MMP is the membrane-associated activator for proMMP-2 in II-$4_{rt}$ cells. The higher molecular protein bands in this zymogram are presumably caused by membrane aggregates containing MT1-MMP, MMP-2 and TIMP-2. Incubation of the membrane fraction with the control antibody anti-HLA-ABC did not result in inhibition of proMMP-2 activation (data not shown).

As both cell types showed active MT1-MMP on their cell surface, although in different amounts, under both culture conditions we asked whether different expression levels of TIMP-2 by HaCaT and II-$4_{rt}$ cells might be critical for proMMP-2 activation. Analysis of TIMP-2 mRNA expression did not reveal significant differences between HaCaT and II-$4_{rt}$ cells grown in collagen gels. (Figure 4). However, the amounts of TIMP-2 protein were strikingly different (Figure 7). Upon contact with fibrillar collagen, TIMP-2 production in II-$4_{rt}$ cells was found to be strongly reduced when compared to monolayer culture, whereas in HaCaT cultures the amount of TIMP-2 increased, resulting in about 6-fold higher levels when compared to II-$4_{rt}$ cells. In contrast, under monolayer conditions both cells types displayed comparable high amounts of TIMP-2 protein. In addition, proMMP-2 activation by membranes isolated from II-$4_{rt}$ cells could be completely inhibited by addition of recombinant TIMP-2 (Figure 6, lanes 6 and 7).

### DISCUSSION

The proteolytic activity of MMP-2 and MMP-9, both degrading type IV collagen in basement membranes, has been proposed to play a critical role for tumour cell invasion which depends on the destruction of tissue barriers.

In a recent study (Meade-Tollin et al, 1998) enhanced expression of these two MMPs was proposed to be associated with acquisition of the tumorigenic phenotype of *c-ras*$^{Ha}$-transfected HaCaT clones. In contrast to this report, we did not detect differences in the amount of latent MMP-2 produced by the non-tumorigenic HaCaT cells and the *c-ras*$^{Ha}$-transfected tumorigenic HaCaT variant II-$4_{rt}$ under comparable culture conditions. However, for MMP-9 the situation was markedly different, showing high amounts of MMP-9 only in the malignant II-$4_{rt}$ cells. This is in good agreement with reports demonstrating that MMP-9 induction is mediated through the *ras* proto-oncogene (Gum et al, 1996).

Upon contact with fibrillar collagen the malignant II-$4_{rt}$ cells, but not the non-tumorigenic HaCaT cells, showed activation of proMMP-2 to its 62/59 kDa active forms. The membrane-associated activation of proMMP-2 was completely abolished in the presence of MT1-MMP-specific antibodies, clearly indicating that other MT-MMPs, although low transcript levels were detected by RT-PCR (data not shown), are not involved in the activation process.

As are all other MMPs, MT1-MMP is synthesized as an inactive zymogen. The mechanisms underlying proMT1-MMP activation and the necessity for cleavage of the N-terminal peptide for its activity are still controversially discussed. MT1-MMP can be activated intracellularly by the protease furin (Pei and Weiss, 1996) and extracellularly by the serine proteases plasmin and urokinase (Okumura et al, 1997; Kazes et al, 1998). However, MT1-MMP-overexpressing COS cells have been shown to activate proMMP-2 without cleavage of proMT1-MMP by furin-like proteases (Cao et al, 1996). In II-$4_{rt}$ cells conversion of latent MMP-2 to the active 62/59 kDa form could be inhibited by the furin inhibitor CMK (Stieneke-Grober et al, 1992), which prevents intracellular activation of proMT1-MMP (Sato et al, 1996; Kurschat et al, 1999). In contrast, the serine protease inhibitors had no effect on the activation process. Furthermore, as we obtained complete inhibition by the furin inhibitor our data convincingly demonstrate that activation of proMT1-MMP by furin is a prerequisite for proMMP-2 activation in II-$4_{rt}$ cells.

Conversion of latent MMP-2 to its active 62/59 kDa forms was observed with II-$4_{rt}$ cells upon growth within collagen gels but not on a collagenous substrate, indicating that activation of proMMP-2 is highly dependent on interactions of the cells with the surrounding matrix. Induction of proMMP-2 activation upon contact with fibrillar collagen type I has also been described for other cell types including fibroblasts (Azzam and Thompson, 1992; Seltzer et al, 1994; Gilles et al, 1997), microvascular endothelial (Haas et al, 1998), and melanoma (Kurschat et al, 1999) cells. In contrast to fibroblasts and endothelial cells, which showed a collagen-induced upregulation of MT1-MMP mRNA, in melanoma cells MT1-MMP transcript levels were barely affected. Instead, melanoma cells showed a collagen-dependent activation of MT1-MMP which, however, did not correlate with the invasive potential of the melanoma cell lines. In our cell system significant amounts of active MT1-MMP protein were detected in all membrane preparations, independent of the tumourigenic phenotype or culture conditions. However, proMMP-2 activation was only obtained with membranes from II-$4_{rt}$ cells grown in collagen gels. This observation strongly suggests that the presence of active MT1-MMP is not sufficient to explain the activation differences observed with HaCaT and II-$4_{rt}$ cells.

As pointed out by many reports, MT1-MMP-mediated activation of proMMP-2 is critically dependent on the amount of TIMP-2 present in the system (Strongin et al, 1995; Will et al, 1996; Butler et al, 1998; Itoh et al, 1998; Zucker et al, 1998). In monolayer cultures TIMP-2 levels were almost comparable in both cell types. However, cultivation in fibrillar collagen induced a dramatic reduction of TIMP-2 protein in II-$4_{rt}$ cells when compared to HaCaT cells. Furthermore, proMMP-2 activation by II-$4_{rt}$ membranes could be completely abolished by an excess of recombinant TIMP-2. These findings indicate that TIMP-2 levels might be critical for the activation process in epidermal cells. Thereby the relative low TIMP-2 levels in II-$4_{rt}$ cells grown in contact with the collagenous matrix might enable MT1-MMP-mediated proMMP-2 activation, whereas the high levels in HaCaT cells might be inhibitory for this process. Addition of recombinant TIMP-1, which has been shown to be a poor inhibitor of proMMP-2 activation (Will et al, 1996), had no effect on proMMP-2 activation (data not shown).

Our study indicates that activation of proMMP-2 rather than enhanced expression might be associated with acquisition of the tumourigenic phenotype in epidermal cells. However, differences in the malignancy of cells might not become obvious unless such more complex in vitro culture systems as three-dimensional collagen lattices are used.

© 2000 Cancer Research Campaign

### ACKNOWLEDGEMENTS

We are grateful to Drs H Sato, N Fusenig and V Magdolen for providing materials and to Dr B Eckes for critical reading of the manuscript. This work was supported by Bundesministerium für Bildung, Wissenschaft, Forschung und Technologie, grants 01 GB 9703/6 and 01 KS 9502/10, and by Deutsche Forschungsgemeinschaft, grant KR 558/10–1.

### REFERENCES

Azzam HS and Thompson EW (1992) Collagen-induced activation of the Mr 72,000 type IV gelatinase in normal and malignant fibroblastoid cells. *Cancer Res* **52**: 4540–4544

Birkedal-Hansen H, Moorie WGI, Bodden MK, Birkedal-Hansen B, DeCarlo A and Engler JA (1993) Matrix metalloproteinases: a review. *Crit Rev Oral Biol Med* **4**: 197–250

Boukamp P, Petrusevska RT, Breitkreutz D, Hornung J, Markham A and Fusenig NE (1988) Normal keratinization in a spontaneously immortalized aneuploid human keratinocyte cell line. *J Cell Biol* **106**: 761–771

Boukamp P, Stanbridge EJ, Foo DY, Cerutti PA and Fusenig NE (1990) c-Ha-ras oncogene expression in immortalized human keratinocytes (HaCaT) alters growth potential in vivo but lacks correlation with malignancy. *Cancer Res* **50**: 2840–2847

Breitkreutz D, Schoop VM, Mirancea N, Baur M, Stark H-J and Fusenig NE (1998) Epidermal differentiation and basement membrane formation by HaCaT cells in surface transplants. *Eur J Cell Biol* **75**: 273–286

Brown PD, Levy AT, Margulies IM, Liotta LA and Stetler-Stevenson WG (1990) Independent expression and cellular processing of $M_r$ 72,000 type IV collagenase and interstitial collagenase in human tumorigenic cell lines. *Cancer Res* **50**: 6184–6191

Butler GS, Will H, Atkinson SJ and Murphy G (1997) Membrane-type-2 matrix metalloproteinase can initiate the processing of progelatinase A and is regulated by the tissue inhibitors of metalloproteinases. *Eur J Biochem* **244**: 653–657

Butler GS, Butler MJ, Atkinson SJ, Will H, Tamura T, Schade van Westrum S, Crabbe T, Clements J, d'Ortho MP and Murphy G (1998) The TIMP-2 membrane type metalloproteinase receptor regulates the concentration and efficient activation of progelatinase A. *J Biol Chem* **273**: 871–880

Cao J, Rehemtulla A, Bahou W and Zucker S (1996) Membrane-type matrix metalloproteinase 1 activates pro-gelatinase A without furin cleavage of the N-terminal domain. *J Biol Chem* **271**: 30174–30180

Carlson SG, Fawcett TW, Bartlett JD, Bernier M and Holbrook NJ (1993) Regulation of the C/EBP related gene gadd153 by glucose deprivation. *Mol Cell Biol* **13**: 4736–4744

Collier IE, Wilhelm SM, Eisen AZ, Marmer BL, Grant GA, Seltzer JL, Kronberger, He C, Bauer EA and Goldberg GI (1988) H-ras oncogene-transformed human bronchial epithelial cells (TBE-1) secrete a single metalloprotease capable of degrading basement membrane collagen. *J Biol Chem* **263**: 6579–6587

Gilles C, Polette M, Seiki M, Birembaut P and Thompson EW (1997) Implication of collagen type I-induced membrane-type 1 matrix metalloproteinase expression and matrix metalloproteinase-2 activation in the metastatic progression of breast carcinomas. *Lab Invest* **76**: 651–660

Gum R, Lengyel E, Juarez J, Chen JH, Sato H, Seiki M and Boyd D (1996): Stimulation of 92-kDa gelatinase B promoter activity by ras is mitogen-activated protein kinase 1 independent and requires multiple transcription factor binding sites including closely spaced PEA3/ets and AP-1 sequences. *J Biol Chem* **271**: 10672–10680

Haas TI, Davis SJ and Madri JA (1998) Three-dimensional type I collagen lattices induce coordinate expression of matrix metalloproteinase MT1-MMP and MMP-2 in microvascular endothelial cells. *J Biol Chem* **273**: 3604–3610

Herron GS, Benda MJ, Clark EJ, Gavrilovic J and Werb Z (1986) Secretion of metalloproteinases by stimulated capillary endothelial cells. *J Biol Chem* **261**: 2814–2818

Itoh Y, Ito A, Iwata K, Tanazawa K, Mori Y and Nagase H (1998) Plasma membrane-bound tissue inhibitor of metalloproteinases (TIMP)-2 specifically inhibits matrix metalloproteinase 2 (gelatinase A) activation on the cell surface. *J Biol Chem* **273**: 24360–24367

Kazes I, Delarue F, Hagege J, Bouzhir-Sima L, Rondeau E, Sraer JD and Nguyen G (1998) Soluble latent membrane-type 1 matrix metalloprotease secreted by mesangial cells is activated by urokinase. *Kidney Int* **54**: 1976–1984

Kurschat P, Zigrino P, Nischt R, Breitkopf K, Steurer P, Klein EC, Krieg T and Mauch C (1999) Tissue inhibitor of matrix metalloproteinase-2 regulates matrix metalloproteinase-2 activation by modulation of membrane-type 1 matrix metalloproteinase activity in high and low invasive melanoma cells. *J Biol Chem* **274**: 21056–21062

Kusukawa J, Sasaguri Y, Shima I, Kameyama T and Morimatsu M (1993) Expression of matrix metalloproteinase-2 related to lymph node metastasis of oral squamous cell carcinomas. A clinicopathologic study. *Am J Clin Pathol* **99**: 18–23

Lehti K, Lohi J, Valtanen H and Keski-Oja J (1998) Proteolytic processing of membrane-type 1 matrix metalloproteinase is associated with gelatinase A activation at the cell surface. *Biochem J* **334**: 345–353

Maquoi E, Noel A, Frankenne F, Angliker H, Murphy G and Foidart JM (1998) Inhibition of matrix metalloproteinase-2 maturation and HT 1080 invasiveness by a synthetic furin inhibitor. *FEBS Lett* **242**: 262–266

Mauch C, Hatamochi A, Scharffetter K and Krieg T (1988) Regulation of collagen synthesis in fibroblast within a three-dimensional collagen gel. *Exp Cell Res* **178**: 493–503

Meade-Tollin LC, Boukamp P, Fusenig NE, Bowen CPR, Tsang TC and Bowden GT (1998) Differential expression of matrix metalloproteinases in activated c-ras$^{Ha}$-transfected immortalized human keratinocytes. *Br J Cancer* **77**: 724–730

Nakamura H, Ueno H, Yamashita K, Shimada T, Yamamoto E, Noguchi M, Fujimoto N, Sato H, Seiki M and Okadea Y (1999) Enhanced production and activation of progelatinase A mediated by membrane-type 1 matrix metalloproteinase in human papillary thyroid carcinomas. *Cancer Res* **59**: 467–473

Okada A, Beloqc JP, Rouyer N, Chenard MP, Rio MC, Chambon P and Basset P (1995) Membrane-type matrix metalloproteinase (MT-MMP) gene is expressed in stromal cells of human colon, breast, head and neck carcinomas. *Proc Natl Acad Sci USA* **92**: 2730–2734

Okumura Y, Sato H, Seiki M and Kido H (1997) Proteolytic activation of the precursor of membrane-type 1 matrix metalloproteinase by human plasmin. *FEBS Lett* **402**: 181–184

Pei D (1999) Identification and characterization of the fifth membrane-type matrix metalloproteinase MT5-MMP. *J Biol Chem* **274**: 8925–8933

Pei D and Weiss SJ (1996) Transmembrane-deletion mutants of the membrane-type matrix metalloproteinase-1 process progelatinase A and express intrinsic matrix-degrading activity. *J Biol Chem* **271**: 9135–9140

Puente XS, Pendas AM, Ilano E, Velasco G and Lopez-Otin C (1996) Molecular cloning of a novel membrane-type matrix metalloproteinase from a human breast carcinoma. *Cancer Res* **56**: 944–949

Pyke C, Ralfkiaer EH, Huhtala P, Hurskaninen T, Dano K and Tryggvason K (1992) Localization of messenger RNA for Mr 72,000 and 92,000 type IV collagenases in human skin cancers by in situ hybridization. *Cancer Res* **52**: 1336–1241

Sato H, Takino T, Okada Y, Shinagawa A, Yamamoto E and Seiki M (1994) A matrix metalloproteinase expressed on the surface of invasive tumor cells. *Nature* **370**: 61–65

Sato H, Takino T, Kinoshita T, Imai K, Okada Y, Stetler-Stevenson WG and Seiki M (1996) Cell surface binding and activation of gelatinase A induced by expression of membrane-type matrix metalloproteinase (MT1-MMP). *FEBS Lett* **385**: 238–240

Seltzer JL, Lee AY, Akers KT, Sudbeck B, Southon EA, Wayner EA and Eisen AZ (1994) Activation of 72-kDa type IV collagenase/gelatinase by normal fibroblasts in collagen lattices is mediated by integrin receptors but is not related to lattice contraction. *Exp Cell Res* **213**: 365–374

Stetler-Stevenson WG, Brown PD, Onisto M, Levy AT and Liotta LA (1990) Tissue inhibitor of metalloproteinase-2 (TIMP-2) mRNA expression in tumor cell lines and human tumor tissues. *J Biol Chem* **265**: 13933–13938

Stetler-Stevenson WG, Aznavoorian S and Liotta LA (1993) Tumor cell interaction with the extracellular matrix during invasion and metastasis. *Annu Rev Cell Biol* **9**: 541–573

Stieneke-Grober A, Vey M, Angliker H, Shaw E, Thomas G, Roberts C, Klenk HD and Garten W (1992) Influenza virus hemagglutinin with multibasic cleavage site is activated by furin, a subtilisin-like endoprotease. *EMBO J* **11**: 2407–2414

Strongin AY, Collier I, Banniko G, Marmer BL, Grant GA and Goldberg GI (1995) Mechanism of cell surface activation of 72-kDa type IV collagenase. Isolation of the activated form of the membrane metalloprotease. *J Biol Chem* **270**: 5331–5338

Takino T, Sato H, Shinagawa A and Seiki M (1995) Identification of the second membrane-type matrix metalloproteinase (MT-MMP2) gene from a human placenta cDNA library. MT-MMPs form a unique membrane-type subclass in the MMP family. *J Biol Chem* **270**: 23013–23020

 © 2000 Cancer Research Campaign

Tomakidi P, Mirancea N, Fusenig NE, Herold-Mende C, Bosch FX and Breitkreutz D (1999) Defects of basement membrane and hemidesmosome structure correlate with malignant phenotype and stromal interactions in HaCaT-Ras xenografts. *Differentiation* **64**: 263–275

Tsunezuka Y, Kinoh H, Takino T, Watanabe Y, Okada Y, Shinagawa A, Sato H and Seiki M (1996) Expression of membrane-type matrix metalloproteinase-1 (MT1-MMP) in tumor cells enhances pulmonary metastasis in an experimental metastasis assay. *Cancer Res* **56**: 5678–5683

Will H and Hinzmann B (1995) cDNA sequence and mRNA distribution of a novel human matrix metalloproteinase with a potential transmembrane domain. *Eur J Biochem* **231**: 602–608

Will H, Atkinson SJ, Butler GS, Smith B and Murphy G (1996) The soluble catalytic domain of type 1 matrix metalloproteinase cleaves the propeptide of progelatinase A and initiates autoproteolytic activation. *J Biol Chem* **271**: 17119–17123

Yu AE, Hewitt RE, Kleiner DE and Stetler-Stevenson WG (1997) Molecular regulation of cellular invasion – role of gelatinase A and TIMP-2. *Biochem Cell Biol* **74**: 823–831

Zucker S, Drews M, Conner C, Foda HD, DeClerk YA, Langley KE, Bahou WF, Docherty AJP and Cao J (1998) Tissue inhibitor of metalloproteinase- (TIMP-2) binds to the catalytic domain of the cell surface receptor, membrane type 1-matrix metalloproteinase 1 (MT1-MMP). *J Biol Chem* **273**: 1216–1222

© 2000 Cancer Research Campaign 
